# *Astragalus cicer* L. and *Astragalus glycyphyllos* L. Flowers as Potential Functional Foods: Nutritional Composition, Chemical Characterization, and Biological Activity

**DOI:** 10.3390/plants15192922

**Published:** 2026-09-24

**Authors:** Saba Shahrivari-Baviloliaei, Liege Aguiar Pascoalino, Filipa S. Reis, Spyridon A. Petropoulos, Agnieszka Konopacka, Pawel Konieczynski, Ilkay Erdogan Orhan, Alina Plenis, Agnieszka Viapiana

**Affiliations:** 1 Department of Analytical Chemistry, Faculty of Pharmacy, Medical University of Gdansk, 80-210 Gdansk, Poland; pawel.konieczynski@gumed.edu.pl (P.K.); alina.plenis@gumed.edu.pl (A.P.); 2 Centro de Investigação de Montanha (CIMO), Instituto Politécnico de Bragança, Campus de Santa Apolónia, 5300-253 Bragança, Portugal; liegeaguiar@ipb.pt (L.A.P.); freis@ipb.pt (F.S.R.); 3 Laboratório Associado para a Sustentabilidade e Tecnologia em Regiões de Montanha (SusTEC), Instituto Politécnico de Bragança, Campus de Santa Apolónia, 5300-253 Bragança, Portugal; 4 Department of Agriculture, Crop Production and Rural Environment, University of Thessaly, Fytokou Street, 38446 Volos, Greece; petropoulos@uth.gr; 5 Department of Pharmaceutical Microbiology, Faculty of Pharmacy, Medical University of Gdansk, 80-210 Gdansk, Poland; agnieszka.konopacka@gumed.edu.pl; 6 Department of Pharmacognosy, Faculty of Pharmacy, Lokman Hekim University, Ankara 06510, Türkiye; ilkay.erdoganorhan@lokmanhekim.edu.tr

**Keywords:** Fabaceae, phenolic composition, minerals, antioxidant activity, antibacterial activity

## Abstract

Investigations into the chemical composition and biological properties of *Astragalus* flowers remain limited. In this context, the present study aimed to investigate the nutritional profile, chemical composition, and in vitro biological activities of *Astragalus cicer* and *Astragalus glycyphyllos* hydroethanolic flower extracts. *A. cicer* flowers had a significantly higher content of ash and crude fats, whereas *A. glycyphyllos* flowers were richer in carbohydrates. The fatty acid profiles varied between the two species, while among tocopherols, only α-tocoferol was detected in both species. Moreover, ten phenolic compounds were identified, with cinnamic acid and rutin being the most abundant phenolic constituents in *A. cicer* and *A. glycyphyllos* flowers, respectively. Mineral analysis revealed that K and Ca were present at the highest levels. Antioxidant activity was determined using the DPPH, FRAP, ABTS and CUPRAC tests, and *A. cicer* flowers were characterized by higher antioxidant activity than *A. glycyphyllos* flowers. Regarding antibacterial activity, growth inhibition zones were observed only for *Staphylococcus aureus,* with diameters ranging from 12 to 14 mm. In conclusion, the results indicate the potential of *A. cicer* and *A. glycyphyllos* flowers as nutritional resources and important dietary sources of natural antioxidants.

## 1. Introduction

Plant flowers have a long history of use as food and for cosmetic and medicinal purposes in various cultures due to their sensory benefits, high nutritional value, and wide range of biological activities [1,2]. Nowadays, they have gained considerable attention from consumers and the market due to these attributes [3]. Their consumption depends on consumers’ individual preferences, as well as on the aroma and health-promoting effects of their chemical components [4]. Generally, edible flowers have a chemical composition similar to that of other plant foods, being characterized by a high water content, low contents of fat and protein, and varying contents of carbohydrates and minerals [5]. They contain various phytochemicals such as flavonoids and phenolic acids, which are potent antioxidants [6]. In addition to their antioxidant activity, phenolic compounds have many physiological functions in the human body, including antimicrobial, anti-inflammatory, and anticancer properties [7].

The genus *Astragalus* L., with approximately 3000 identified species, is one of the largest genera of flowering plants in the Fabaceae family and is widely distributed in arid and continental regions of Asia, America, and Africa. This genus has been used in folk medicine for over 2000 years, particularly in countries such as China, Türkiye, and Iran, due to its various therapeutic properties [8]. The leaves and roots of *Astragalus* species are widely used as analgesic, anticancer, antidiarrheal, anti-infective, and anti-inflammatory agents [8], while the flowers are traditionally consumed as tea and exhibit efficient antioxidant activity [9]. *Astragalus* plants contain bioactive compounds, which exhibit various pharmacological activities, including antioxidant, anti-inflammatory, and antimicrobial properties [10,11]. Owing to these activities, *Astragalus* species can be considered promising herbal raw materials for health-related products.

*Astragalus cicer* L. and *Astragalus glycyphyllos* L. have been used in traditional medicine as well as for food purposes in several European countries [12,13]. In France, the herbs of *A. glycyphyllos* are used as an emollient, diuretic, and refreshing agent [13]. Previous research has indicated hypotensive, anticoagulant, and diuretic activities of 10% infusions of the aerial part of *A. glycyphyllos* [14], while in Belarus, *A. cicer* is used to treat heart and gastrointestinal diseases [15]. The flowers of *A. glycyphyllos* are up to 16 mm long, and the bracts are lanceolate, with sparsely ciliate white edges. The calyx is 5–6 mm long, glabrous or loosely covered with appressed black hairs, and the petals are up to 15 mm long and yellowish-green [16]. In the case of *A. cicer*, inflorescences grow in racemes and contain 15 to 60 flowers, which are pale yellow and 15–16 mm in length, asympetalous, and have a five-lobed hairy calyx [17].

Astragali Radix (the dried root of *Astragalus membranaceus*) is a medicinal herb belonging to the same genus that has been extensively researched. It contains a variety of active ingredients, among which saponins, polysaccharides and flavonoids have been reported most frequently. All these types of ingredients have some therapeutic effects, including effects on type 2 diabetes mellitus (T2DM) [18,19,20]. The primary effects of *Astragalus* polysaccharides on diabetes include a reduction in insulin resistance, stimulation of islet cell proliferation, and suppression of islet β-cell death [18]. Additionally, *Astragalus* polysaccharides have a positive regulatory effect on the intestinal microbiota and exhibit antifibrosis pharmacological activity [21], while flavonoids have immune-boosting, anti-inflammatory, glucose metabolism-regulating, and diabetes progression-controlling properties [18]. In the case of *Astragalus* flowers, previous studies indicate that they contain relatively high levels of flavonoids, triterpenoid saponins, phenolic acids, and volatile compounds [22]. However, investigations into the chemical composition and biological properties of *Astragalus* flowers are still very limited. In this context, the present study aimed to investigate the nutritional profile, chemical composition, and in vitro bioactivities of *A. cicer* and *A. glycyphyllos* flowers, assessed through antioxidant capacity and antibacterial activity assays against *Staphylococcus aureus* and *Escherichia coli.* Assays for free radical scavenging and reducing power were employed to assess their antioxidant capacity. The total phenol and flavonoid contents, as well as individual phenol and flavonoid compounds, in the extracts of *Astragalus* flowers were also investigated, aiming to demonstrate the possibility of utilizing these flowers as a novel by-product in pharmaceutical, food, or personal care applications. To our knowledge, this is the first comprehensive study of the chemical profiles of *A. cicer* and *A. glycyphyllos* flowers. Moreover, the present study not only provides novel data on the phytochemical properties of these species but also highlights their antimicrobial and antioxidant potentials. Ultimately, this study aims to expand current knowledge of the potential of the flowers of these *Astragalus* species, supporting future research and practical applications in food technology, functional product development, and the nutraceutical industry.

## 2. Results and Discussion

### 2.1. Nutritional Composition, Energetic Value and Free Sugars Profile

The nutritional composition of *A. cicer* and *A. glycyphyllos* flowers is presented in Table 1. The species did not differ significantly in total protein content (20.12 and 19.92 g/100 g dw, for *A. cicer* and *A. glycyphyllos*, respectively) or energetic value (381 and 380 kcal/100 g dw) for *A. cicer* and *A. glycyphyllos*, respectively. In contrast, *A. cicer* had significantly higher contents of ash and crude fats (7.91 and 2.59 g/100 g dw, respectively), whereas *A. glycyphyllos* flowers were richer in carbohydrates (71.03 g/100 g dw). According to the literature, *Astragalus* species are a rich source of protein, especially the seeds of *A. caprinus* and *A. armatus,* which may contain up to 54% crude protein [23]. Regarding other plant organs, the fruits of *A. cicer* and *A. glycyphyllos* are also protein-rich, displaying higher levels than the roots and leaves (26.1% and 19.6% protein in the fruits of *A. cicer* and *A. glycyphyllos*, respectively) [24]. Apart from the plant part, protein content may also vary depending on the harvesting stage, since its content decreases with maturity as plants enter the flowering stage [25]. Furthermore, macronutrient composition varies depending on the plant organ, which is consistent with the findings reported by Shahrivari-Baviloliaei et al. [24] for the leaves, roots and fruits of *A. cicer* and *A. glycyphyllos*. To the best of our knowledge, this is the first report regarding the nutritional characterization of the flowers of *A. cicer* and *A. glycyphyllos,* and our results highlight their high nutritional value and potential use as edible flowers. However, additional safety studies are necessary to ensure the absence of potentially toxic compounds.

Regarding soluble sugars, sucrose, fructose, and glucose were detected in both species (Table 1). However, although the total sugar content did not differ between the two species, the amounts of individual compounds showed significant differences, especially in the case of fructose and sucrose, for which *A. glycyphyllos* and *A. cicer* recorded the highest values (5.40 g/100 g dw and 6.90 g/100 g dw, for fructose and sucrose, respectively). *Astragalus* species are rich in carbohydrates, mainly because of their polysaccharides and mucilage, but the exact content is highly dependent on the species and analytical method; reported values range from very low levels to above 80–90% sugar content after isolation [26,27]. Moreover, the composition of saccharides may differ depending on the species and the plant part, as indicated by Niknam and Salehi-Lisar [26], who reported glucose as the major compound in several *Astragalus* species, while fructose, galactose, and glucuronic acid were the most abundant compounds in *A. semihinatus*, *A. monspessulanus* spp., and *A. aegobromus* Boiss. and Hohen., respectively. Furthermore, Shahrivari-Baviloliaei et al. [24] detected a varied sugar profile in the roots, leaves, and fruits of *A. cicer* and *A. glycyphyllos*, with fructose being the major free sugar in the leaves of both species, while sucrose was most abundant in the roots. The fruits contained low levels of free sugars, with fructose and sucrose identified in higher amounts in *A. glycyphyllos* and *A. cicer*, respectively.

Table 2 outlines the fatty acid profile, which varied between the two species. In particular, 12 individual fatty acids were detected in *A. cicer*, whereas seven compounds were observed in *A. glycyphyllos*. Moreover, apart from the different number of compounds detected, the major compounds in *A. cicer* were oleic acid (29.00%), followed by palmitic acid (19%), linoleic acid and α-linolenic acid (18.30 and 17.41%, respectively), while stearic, erucic, nervonic, and tricosylic acids were detected in lesser amounts (4.90%, 3.51%, 1.90%, and 1.40%, respectively). On the other hand, in *A. glycyphyllos*, the major compounds were α-linolenic acid (26.80%), linoleic acid (22.20%), palmitic acid (21.90%) and arachidic acid (13.50%), followed by stearic, oleic, and myristic acids (9.60%, 4.50% and 1.59%, respectively). *A. glycyphyllos* exhibited comparable proportions of polyunsaturated fatty acids (PUFA) and saturated fatty acids (SFA) (49.00% and 46.56%, respectively), which accounted for approximately 95% of total fatty acids. The fatty acid profile of *A. cicer* was predominantly composed of MUFAs (35.01%) and PUFAs (35.70%), alongside a notable presence of SFAs (29.00%). The fatty acid profile of *Astragalus* spp. may vary depending on the species and the plant part [24,28]. A similar trend was recorded by Mahmoudi et al. [23] and Haşimi et al. [29], who also reported a variable fatty acid composition among several *Astragalus* species (*A. leporinus* var. *hirsutus, A. distinctissimus*, and *A. schizopterus*). Moreover, the major compounds were palmitic acid, α-linolenic acid, linoleic acid, and arachidic acid, a profile that was also recorded in the fruits of *A. glycyphyllos* in our study [24], while Klichkhanov et al. [30] reported α-linolenic, palmitic, linoleic and oleic acids as the most abundant fatty acids in *A. glycyphyllos* leaves.

Regarding tocopherols, only α-tocopherol was detected in both species, with *A. glycyphyllos* showing a significantly higher content than *A. cicer* (2.80 and 1.29 mg/100 g dw, respectively) (Table 2). According to Shahrivari-Baviloliaei et al. [24], α-tocopherol was the main vitamin E isoform detected in various parts (leaves, roots, and fruits) of *A. cicer* and *A. glycyphyllos*, especially the leaves, which also contained β- and γ-tocopherol (the latter was detected only in the leaves of *A. glycyphyllos*). Therefore, it could be suggested that, in addition to the plant part, the genotype may also regulate tocopherol biosynthesis and affect its composition in plant tissues. Abdellaoui et al. [31] also detected α-tocopherol in *Astragalus gombiformis* leaves and reported an increase in its content when plants were grown under water-deficit conditions. Additionally, Bahşi et al. [32] observed a variable tocopherol profile among the seeds of five *Astragalus* species, although α-tocopherol was consistently detected across all samples, whereas β- and γ-tocopherol were only detected in *A. anthloides*, *A. leporinus* var. *hirsutus*, and *A. campylorhunchus*.

### 2.2. Total Phenolic, Flavonoid, Phenolic Acid, and Tannin Contents

The spectrophotometric method is frequently used to estimate the total phenolic content in plant material samples. In this study, TPC, TFC, TPAC, and TAN in the flowers of both *Astragalus* species were determined, and the results are shown in Table 3. Moreover, an ANOVA was carried out to compare the phenolic, flavonoid, phenolic acid and tannin contents between the two plant species. Generally, *A. cicer* extracts had significantly higher TPC (*p* < 0.05) than the extracts of *A. glycyphyllos,* while *A. glycyphyllos* extracts were significantly richer (*p* < 0.05) in TFC. Moreover, there were no significant differences between *A. glycyphyllos* and *A. cicer* flowers for the other parameters. According to the literature, in the *A. glycyphyllos* flowers from Greece, Butkute et al. [33] reported TPC and TFC levels of 23.71 mg GAE/g dw and 16.71 mg RE/g dw, respectively. The high phenolic content detected in these floral extracts points to significant antioxidant capacity. Such polyphenolic compounds are of broad interest, as epidemiological studies indicate they may offer protection against chronic conditions, including cancer, osteoporosis, cardiovascular diseases, diabetes, and neurological disorders [34]. However, differences in phenolic compound profiles can be driven by multiple factors, such as the plant’s age, genotype, growing conditions, location, the solvent used in extraction, and harvesting methods [34,35]. Such parameters likely contributed to the differences in phenolic content observed in the present study.

With respect to flowers from other species of the genus *Astragalus*, Lekmine et al. [36] analyzed the flowers of *A. gombiformis* from Algeria and reported a TPC level of 99.304 ppm. Aydemir et al. [37] found TPC and TFC levels of 130.27 mg GAE/g and 66.85 mg QE/g in methanolic extracts of Turkish *A. gymnolobus* Fisch flowers. Moreover, Doğan et al. [38] analyzed the flowers of Turkish *A. chamaepnaca* Freyn using water, methanol and ethanol as extraction solvents and found TPC levels of 713.178, 579.497 and 631.783 µg GAE/g, respectively. Similarly, Comlekcioglu et al. [39] evaluated *A. angustifolius* flowers of Turkish origin prepared in different extraction solvents and found TPC and TFC levels of approximately 4.84 mg GAE/g and 5.02 µg QE/g, respectively, in methanolic extracts, while lower TPC and TFC values were obtained in ethanolic extracts (1–2 mg GAE/g and 0.5–1 µg QE/g, respectively). These findings demonstrate that solvent selection plays an important role in the extraction yield of phenolics and flavonoids from *Astragalus* flowers. To the best of our knowledge, no published data are available regarding TPAC and TAN in the flowers of *A. glycyphyllos* and TPC, TFC, TPAC, and TAN in the flowers of *A. cicer*.

### 2.3. Profiling Individual Phenolic Compounds

Regarding the phenolic composition of the extracts, ten phenolic compounds were identified and quantified, including five phenolic acids [gallic (GA), protocatechuic (PTA), ferulic (FA), rosmarinic (RA) and cinnamic (CNA) acids] and five flavonoids [rutin (RUT), quercetin (Q), genistein (GENIS), formononetin (FORM) and biochanin (BIOCH)] (Table 3). The obtained results were expressed as mg or µg per g of plant material on a dry weight (dw) basis. Significant differences were observed in the levels of individual phenolic compounds between *A. cicer* and *A. glycyphyllos* flower extracts. However, CNA was the most abundant phenolic constituent in *A. cicer* flowers, while RUT was the most abundant phenolic constituent in *A. glycyphyllos* flowers, whereas the levels of GENIS, BIOCH and PTA were the lowest in this species. FORM was not detected in any of the tested samples. On the other hand, CNA was the most abundant phenolic compound in *A. cicer* flowers, while RA and Q were present at higher levels in *A. glycyphyllos* flowers. To the best of our knowledge, this is the first report on individual phenolic compounds in *A. cicer* and *A. glycyphyllos* flowers. Regarding other species of *Astragalus*, Lekmine et al. [36] determined a Q content of 0.977 ppm in *A. gombiformis* flowers from Algeria, while GA, PTA and FA were not detected. Doğan et al. [38] detected GA, FA, RUT and Q in aqueous, methanolic, and ethanolic extracts of *A. chamaephaca* Freyn from Türkiye and found the highest level of GA in the aqueous extract of flowers (204.309 µg/g). Conversely, GA levels in the remaining extracts were either below the detection limit or extremely low (below 40 µg/g). Moreover, the levels of RUT and FA ranged from 24.811 µg/g in the aqueous extract to 99.917 µg/g in the methanolic extract, and from 11.538 µg/g in the methanolic extract to 27.43 µg/g in the aqueous extract, respectively. Finally, the level of Q ranged from 0.540 µg/g in the aqueous extract to 7.49 µg/g in the ethanolic extract. Aydemir et al. [37] also determined phenolic compounds in flower methanol extract of *A. gymnolobus* from Türkiye and found FA and Q at the concentrations of 24.9 µg/g and 6.07 µg/g, respectively. These differences in phenolic compound content compared to our study could be attributed to differences in the extraction methodology, as well as to differences in genotype and growing conditions [40].

### 2.4. Mineral Profiling

The availability of micro- and macroelements in plants plays an important role in the biosynthesis of phenolic compounds and other secondary metabolites. Macroelements such as K, Ca and Mg are involved in the regulation of enzymatic activity, photosynthesis, and energy metabolism, while micronutrients such as Fe and Zn act as cofactors for enzymes involved in the synthesis of flavonoids and other polyphenolic compounds [41]. Knowledge of the levels of elements in plants is important because many of them play a crucial role in the formation of constituents responsible for their therapeutic and nutritional properties. The uptake of elements by plants primarily occurs through two main pathways: root uptake and foliar uptake. Root uptake involves the absorption of elements from the soil solution and is influenced by various factors such as soil pH, metal speciation, organic matter content, and root characteristics [42]. In turn, foliar uptake involves the direct absorption of some elements from the atmosphere through the stomata or their deposition onto the plant’s surface [43]. Moreover, the botanical species may also affect the concentration of these elements in plants. In the case of herbal formulations, elements may be present as a result of bioaccumulation at the harvest site or introduced during postharvest processes such as grinding, drying, and solvent extraction.

In the current study, four trace elements (Zn, Fe, Pb, and Cd) and four macronutrients (K, Na, Ca, and Mg) were determined, and the results from the element analysis of hydroethanol extracts prepared from *A. cicer* and A. *glycyphyllos* flowers are presented in Table 4. Overall, the analyzed *Astragalus* flower extracts displayed comparable elemental contents. Among the macroelements, K and Ca were determined at the highest levels, while among the microelements, only *essential trace elements* (Fe and Zn) were found in the analyzed extracts. In the case of the potentially toxic elements (Cd and Pb), their contents were below the detection limit of the instrument in both *Astragalus* species. Most adults need 1000 mg of Ca and 3500 mg of K per day [44]. Based on our results, he flowers of both *Astragalus* species contain nutritionally important elements such as Ca and K. In the existing literature, elemental analysis of *A. cicer* and *A. glycyphyllos* flowers is limited to the study by Butkute et al. [33], who recorded higher concentrations of K, Ca, Mg, Zn, and Fe than those found in our findings. For instance, K was present at a level of about 3.0 g/100 g and Fe about 13–14 mg/100 g, while in this study, K was determined at a level of about 19 mg/g, and Fe at about 5.0 mg/100 g. Regarding flowers of other species of *Astragalus*, Doğan et al. [38] analyzed *A. chamaephaca* Freyn flowers from Türkiye and found the highest levels of K (17,680.03 mg/L) and Ca (7407.43 mg/L). Moreover, the contents of Mg (604.22 mg/L) and Fe (429.09 mg/L) were also high, while the concentrations of Na and Zn were the lowest, at 28.15 and 10.33 mg/L, respectively. The presence of mineral elements in plants is strongly influenced by the plant species as well as by factors including soil chemistry, watering regimes, and atmospheric contamination [45].

### 2.5. Biological Activity

#### 2.5.1. Antioxidant Activity

Because of the lack of standardized methods for antioxidant capacity evaluation, it is essential to perform more than one assay to evaluate antioxidant capacity and account for the various mechanisms of antioxidant action. Accordingly, the antioxidant profile was evaluated using four complementary assays, namely DPPH, ABTS, FRAP, and CUPRAC. Radical scavenging activity was evaluated using DPPH and ABTS assays, which operate through hydrogen atom transfer (HAT), single electron transfer (SET), or a combination of both mechanisms depending on the reaction conditions and antioxidant structures. The FRAP assay is based exclusively on a SET mechanism, measuring the ability of antioxidants to reduce Fe^3+^ to Fe^2+^ under acidic conditions, whereas the CUPRAC assay measures the reduction of Cu^2+^ to Cu^+^ by antioxidants. These methods are cost-effective, rapid, stable, and selective.

The results of the DPPH, ABTS, FRAP and CUPRAC assays used to determine the antioxidant activity of *A. cicer* and *A. glycyphyllos* flowers were presented in Table 5. Generally, *A. cicer* flowers were characterized by higher antioxidant activity, especially in the FRAP assay, compared to *A. glycyphyllos* flowers. This may be due to the fact that different methods for determining antioxidant capacity have different sensitivities to solvents, reagents, pH conditions, and hydrophilic/hydrophobic substances [46]. Moreover, due to the relationship between the content of phenolic compounds and antioxidant capacity, the higher antioxidant activity of *A. cicer* flowers can be explained by their higher phenolic compound content. Additionally, it may also be a result of the presence of different constituents that were not determined in this study, but may be present in this species. Moreover, no significant differences were found between the DPPH and CUPRAC values obtained for the two *Astragalus* flowers. To the best of our knowledge, no data on the antioxidant activity of *A. cicer* and *A. glycyphyllos* flowers have been reported in the published literature. However, regarding flowers of other species of *Astragalus*, Lekmine et al. [36] reported DPPH and ABTS values for the hydroethanolic extracts of *A. gombiformis* flowers at the levels of 16.43 and 16.13 µg/mL, respectively. Comlekcioglu et al. [35] analyzed the flowers of *A. angustifolius* using different solvents and found DPPH and FRAP values in methanolic extracts ranging from 4.5 to 7.01 µg ascorbic acid/mL (IC_50_) and from 3.61 to 3.90 mg ascorbic acid/g, respectively, while the values in the ethanolic extracts ranged from 4.85 to 7.33 µg ascorbic acid/mL (IC_50_) for DPPH and from 2.36 to 2.43 mg ascorbic acid/g for ABTS. Doğan et al. [38] also determined the antioxidant activity of Turkish *A. chamaephaca* Freyn. flowers using different solvents and obtained DPPH values of 0.421, 0.374 and 0.935 mg/mL (IC_50_) for aqueous, methanolic, and ethanolic extracts, respectively, while the corresponding ABTS values were lower (0.130 mg/mL (IC_50_) for the aqueous extract, 0.098 mg/mL (IC_50_) for the methanolic extract, and 0.158 mg/mL (IC_50_) for the ethanolic extract. These results could not be compared with data obtained in our study due to the difference in presentation methods (results expressed as percentage inhibition of DPPH and ABTS free radicals). In contrast, Aydemir et al. [37] found a high DPPH value (IC_50_: 759 µg/mL) in a methanolic extract of *A. gymnolobus* flowers, while FRAP and CUPRAC values were 0.65 mM FeSO_4_/g and 19 mg AAE/g, respectively.

#### 2.5.2. Antibacterial Activity

The antibacterial activity of hydroethanol extracts from *A. cicer* and *A. glycyphyllos* flowers was tested against *S. aureus* and *E. coli* using the diffusion method on a solid medium. The results are presented in Table 5. At the tested concentration of 300 mg/mL, the diameter of growth inhibition zones was found only for *S. aureus,* with diameters ranging from 12 to 14 mm. Plant polyphenols are generally considered to exhibit antimicrobial activity. Indeed, antimicrobial properties have been documented for various *Astragalus* species [37], likely reflecting variations in both the concentration and specific types of phytochemicals present.

## 3. Materials and Methods

### 3.1. Materials and Reagents

*A. cicer* and *A. glycyphyllos* were collected from the botanical garden of Maria Curie-Sklodowska University in Lublin (Poland) in 2023. The flowers of each plant species were separated, washed with tap water to remove the soil, dried in the shade, and pulverized in a water-cooled grinder (Knifetec 1095, Foss Tecator, Höganäs, Sweden) at 20 °C for 20 s.

4-Chloro-7-nitrobenzofurazan (NBD-Cl), 2,2-diphenyl-1-picrylhydrazyl (DPPH reagent), ten standards, including protocatechuic acid (PTA), gallic acid (GA), rosmarinic acid (RA), cinnamic acid (CNA), ferulic acid (FA), quercetin (Q), rutin (RUT), genistein (GENIS), formononetin (FORM), and biochanin (BIOCH), as well as tocopherols (α-, β-, γ-, and δ-isoforms) were purchased from Sigma-Aldrich (St. Louis, MO, USA) [34]. Other reagents were obtained from POCh (Gliwice, Poland). Redistilled water was prepared as previously described by Shahrivari-Baviloliaei et al. [24]. Racemic tocol (50 mg/mL) was purchased from Matreya (State College, PA, USA). Methanol and hexane purchased from Fisher Scientific (Lisbon, Portugal) were of analytical grade.

### 3.2. Sample Preparation

A powdered sample (0.5 g) was combined with 70% (*v*/*v*) aqueous ethanol (5 mL), and the mixture was extracted for 30 min at 30 °C in an ultrasonic bath (Emag, Salach, Germany). Following extraction, the mixture was centrifuged at 4000× *g* for 10 min using a centrifuge (EBA-20S, Hettich, Tuttlingen, Germany). The resulting supernatant was transferred to a 10 mL volumetric flask. The procedure was repeated twice, and at the end the extracts were filtered using Whatman No. 1 filter paper, combined and diluted to 10 mL with 70% (*v*/*v*) aqueous ethanol. The prepared extracts were stored at −20 °C until further analysis.

Before high-performance liquid chromatography (HPLC) analysis with a UV/Vis detector, the hydroethanolic flower extracts of *A. cicer* and *A. glycyphyllos* were filtered, and 20 µL of each filtered extract was then injected into the HPLC system, as previously described by Polumackanycz et al. [47], while tocopherols were determined according to the procedure previously reported by Sprea et al. [48].

### 3.3. Nutritional Profile

Total protein, crude fat, ash, and carbohydrate contents were determined in the freeze-dried samples, following the methods of the Association of Official Analytical Chemists (AOAC) methods [49]. Protein content was estimated by the macro-Kjeldahl method (model Pro-Nitro-A; Selecta, Barcelona, Spain) (AOAC 920.87); crude fat content was determined by Soxhlet extraction with petroleum ether (AOAC 948.22); the ash content was evaluated by incineration in a muffle furnace at 550 ± 15 °C (AOAC 923.03); total carbohydrates were estimated by difference; and the energy value was calculated using the following formula: energy (kcal/100 g) = 4 × (g protein + g carbohydrates) + 9 × (g fat). Details of the methodologies are described by Pascoalino et al. [50]. The results were presented as g per 100 g of plant material on a dry weight (dw) basis.

### 3.4. Composition in Free Sugars

Free sugars were analyzed using an HPLC system (Knauer, Smartline system 1000, Berlin, Germany) equipped with a refractive index (RI) detector, as previously described by Pascoalino et al. [50]. Sugars were identified and quantified by comparing their retention times with those of authentic reference standards. The results were expressed in g per 100 g of plant material on a dry weight (dw) basis.

### 3.5. Composition in Fatty Acids and Tocopherols

The fatty acid methyl ester (FAME) mixture was analyzed using a YOUNG IN Chromass 6500 Gas Chromatography System (YL6500, Seoul, Republic of Korea) equipped with a flame ionization detector (FID), as previously described [48]. Separation was carried out on a Zebron™ ZB-FAME column (30 m × 0.25 mm, 0.20 μm) using the following oven temperature program: initial temperature: 100 °C, held for 2 min; increase at 10 °C/min until reaching 140 °C; increase at 3 °C/min to 190 °C; and increase at 30 °C/min until reaching 260 °C. Hydrogen was used as the carrier gas at a flow rate of 1.2 mL/min, measured at 250 °C. Identification and quantification were conducted by comparing the retention times of the sample FAME peaks with those of the 47885-U standard (Sigma, St. Louis, MO, USA). Results were processed using Clarity DataApex 4.0 Software (Prague, Czech Republic) and were expressed as the relative percentage of each fatty acid.

Tocopherols were analyzed using an HPLC system, referred to Section 3.4, coupled with a fluorescence detector (FP-2020, Jasco, Easton, MD, USA) set at excitation and emission wavelengths of 290 nm and 330 nm, respectively, following a previously described analytical procedure [48]. Identification and quantification were conducted by comparison with authentic standards using the internal standard method, while the results were expressed as mg per 100 g of plant material.

### 3.6. Determination of Total Phenolic (TPC), Total Flavonoid (TFC), Total Phenolic Acids (TPAC) and Tannins (TAN) Contents

Total phenolic content was determined using the Folin–Ciocalteu method [51], and the results were expressed as mg of gallic acid equivalents (GAE) per g of dry sample (dw). TFC was determined according to the European Pharmacopoeia method [52], and the results were expressed as mg of quercetin equivalents (QE) per g of dw. TPAC content was determined according to the method described in the Polish Pharmacopoeia VI [53], and the results were expressed as μg of caffeic acid equivalents (CAE) per g of dw. TAN content was estimated using the method described by Sun et al. [54], and the results were reported as mg of catechin equivalents (CAT) per g of dw.

### 3.7. Chromatographic Analysis of Individual Phenolic Compounds

The qualitative and quantitative HPLC analyses of phenolic compounds were performed using a Merck-Hitachi LaChrome HPLC system (Darmstadt, Germany) coupled to an L-7420 UV/Vis detector. Chromatographic separations were carried out using a Hypersil Gold C18 column (250 × 4.6 mm, 5 µm particle size) (Thermo Scientific, Runcorn, UK). The column was operated at a constant temperature of 30 °C, the volume of the analyzed extract was 20 µL, and the flow rate was 1 mL/min according to the procedure previously reported by Viapiana et al. [55]. The mobile phase consisted of 0.1% acetic acid in methanol (solvent A) and 0.1% acetic acid in water (solvent B). The gradient program was as follows: 0–10 min, 5–15% A; 10–15 min, 15–20% A; 15–20 min, 20–30% A; 20–30 min, 30–63% A; 30–35 min, 63% A; and 35–40 min, 63–5% A. For the quantitative analysis, the calibration curves were obtained by injecting known concentrations of individual standard compounds, and the phenolic compounds were detected at 280 nm (GA, CNA, PTA, GENIS, BIOCH, and FORM), 320 nm (FA and RA), and 370 nm (RUT and Q). Phenolic compounds were identified by comparing their retention times with those of the corresponding standard compounds. Moreover, a selected sample was spiked with the standard compounds and reanalyzed [47].

The validation parameters for the HPLC procedure are presented in Table S1. The calibration curves for ten phenolic compounds were linear and reproducible, with the correlation coefficients (R^2^) > 0.993. The limit of detection (LOD) and limit of quantification (LOQ) were determined based on the signal-to-noise (S/N) ratio (S/N of 3 for LOD and S/N of 10 for LOQ, respectively). The lowest LOD and LOQ values were obtained for RUT, at 2.2 μg/mL and 9.1 μg/mL, respectively, while the highest values were obtained for FORM, at 3.7 μg/mL and 14.8 μg/mL, respectively. The recovery values ranged from 92.92% to 105.32%, and the relative standard deviation (RSD) was below 5% under repeatability conditions for the studied analytes. The precision was acceptable, with CV values ranging from 0.25 to 4.02% and from 0.32 to 5.21% for intra- and inter-day variations, respectively.

### 3.8. Mineral Profiling

A flame atomic absorption/emission spectrometer (SpectrAA 250Plus, Varian, Australia) was used to determine microelements (Zn, Fe, Pb, and Cd) and macroelements (K, Na, Ca, and Mg). Microelements were determined by atomic absorption spectroscopy, while macroelements were determined by atomic emission spectroscopy. The analytical wavelengths used for the individual elements were as follows: 214.0 nm (Zn), 248.0 nm (Fe), 228.8 nm (Cd), 217.0 nm (Pb), 766.5 nm (K), 589.0 nm (Na), 422.7 nm (Ca), and 285.2 nm (Mg). The analytical procedures were validated, and the results for linearity, recovery, LOD, and LOQ data were deemed satisfactory [56].

### 3.9. Determination of Antioxidant Activity

The antioxidant activity of the *A. glycyphyllos* and *A. cicer* flower extracts was evaluated in vitro using the 2,2-diphenyl-1-picrylhydrazyl (DPPH), ferric reducing antioxidant power (FRAP), 2,2-azino-bis-3-ethylbenzothiazoline-6-sulphonic acid (ABTS), and cupric reducing antioxidant capacity (CUPRAC) assays. The DPPH assay was performed as previously reported [24], and the results were expressed as mg of Trolox equivalents (TE) per g of dw. The FRAP assay was conducted according to the previously reported method in the literature [24], and the results were expressed as mg of ferrous ion equivalents (Fe^2+^) per g of dw. Following the protocol described by Shahrivari-Baviloliaei et al. [24], the ABTS radical scavenging activity was measured and expressed as mg of Trolox equivalents (TE) per g of dw. Finally, the CUPRAC assay was performed according to the method previously described in the literature, and the results were expressed as mg of ascorbic acid equivalents (AA) per g of dw [24].

### 3.10. Determination of Antibacterial Activity

The antimicrobial potential of the *A. cicer* and *A. glycyphyllos* flower extracts was preliminarily evaluated using an agar well diffusion test. For this study, *S. aureus* ATCC 6538 and *E. coli* ATCC 8739 strains were used according to the procedure described by Shahrivari-Baviloliaei et al. [24]. The diameters of the growth inhibition zones were measured, with ampicillin used as the reference antibiotic.

### 3.11. Data Analysis

The results obtained in this study are expressed as means and standard deviations (SDs). Statistical analysis was performed using Statistica 10 software (StatSoft Inc., Tulsa, OK, USA). One-way ANOVA was used to assess differences among the analyzed samples, followed by Tukey’s honestly significant difference (HSD) test at *p* < 0.05. All assays were performed in triplicate in three independent experiments for each *Astragalus* species.

## 4. Conclusions

The present study provides a comprehensive analysis of the nutritional, chemical and biological properties of hydroethanolic extracts prepared from the flowers of *A. cicer* and *A. glycyphyllos*. To our knowledge, this study provides the first evidence of the biological activities of the flowers of these two *Astragalus* species. *A. cicer* flowers demonstrated significantly higher concentrations of ash, crude fats, individual phenolic compounds (GA, FA, CNA, GENIS, BIOCH), TPC, and also higher values of the FRAP assay, while *A. glycyphyllos* flowers exhibited higher levels of carbohydrates, individual phenolic compounds (PTA, RUT, RA, Q), TFC, and also the value of the ABTS assay. Moreover, the flower extracts of *A. cicer* and *A. glycyphyllos* flowers were particularly rich in K and Ca, whereas their antibacterial activity was observed exclusively against *S. aureus*. These findings highlight the nutritional and functional potential of both *Astragalus* species, particularly *A. cicer* flowers, which consistently exhibited higher levels of bioactive compounds and antioxidant activity and were also rich in several nutrients. In conclusion, the results indicate that *A. cicer* and *A. glycyphyllos* flowers may represent a valuable resource for the development of health-promoting food products and dietary sources of natural antioxidants. However, this study did not assess the bioavailability of the identified compounds, the effects of processing on their bioactive compounds and nutritional quality, or their sensory attributes. Further studies addressing these aspects are needed to support the potential use of these flowers as functional foods and ingredients for human consumption.

## Figures and Tables

**Table 1:** Nutritional and energetic value, and soluble sugars of the flowers of *A. cicer* and *A. glycyphyllos* (mean ± SD).

	*A. cicer* Flowers	*A. glycyphyllos* Flowers
**Nutritional value** (g/100 g dw)		
Total protein	20.12 ± 0.02 ^a^	19.92 ± 0.23 ^a^
Ash	7.91 ± 0.23 ^a^	7.31 ± 0.41 ^b^
Crude fats	2.59 ± 0.05 ^a^	1.80 ± 0.12 ^b^
Carbohydrate content	69.42 ± 0.11 ^b^	71.03 ± 0.30 ^a^
Total energy (kcal/100 g)	381 ± 1.04 ^a^	380 ± 1.05 ^a^
**Soluble sugars** (g/100 g dw.)		
Fructose	3.52 ± 0.31 ^b^	5.40 ± 0.10 ^a^
Glucose	2.11 ± 0.22 ^a^	1.92 ± 0.10 ^a^
Sucrose	6.90 ± 0.30 ^a^	4.80 ± 0.41 ^b^
Unidentified	0.51 ± 0.00 ^a^	0.91 ± 0.00 ^b^
Total	13.04 ± 1.01 ^a^	13.03 ± 1.03 ^a^

Means in the same row followed by different letters are significantly different according to Tukey’s honestly significant difference (HSD) test at *p* < 0.05.

**Table 2:** Composition of fatty acids (relative %) and of tocopherols (mg/100 g dw) of the studied flowers of *A. cicer* and *A. glycyphyllos* (mean ± SD, n = 3).

	*A. cicer* Flowers	*A. glycyphyllos* Flowers
C8:0	nd	nd
C12:0	nd	nd
C14:0	nd	1.59 ± 0.02
C15:0	0.37 ± 0.01	nd
C16:0	19.00 ± 1.01 ^b^	21.90 ± 0.61 ^a^
C16:1	0.57 ± 0.01	nd
C17:0	0.59 ± 0.04	nd
C17:1	nd	nd
C18:0	4.90 ± 0.1 ^b^	9.60 ± 0.10 ^a^
C18:1n9t	nd	nd
C18:1n9c	29.00 ± 2.01 ^a^	4.50 ± 0.30 ^b^
C18:2n6c	18.30 ± 0.50 ^b^	22.20 ± 0.10 ^a^
C18:3n3	17.41 ± 0.40 ^b^	26.80 ± 0.30 ^a^
C20:0	2.90 ± 0.10 ^a^	13.50 ± 0.30 ^b^
C20:1	nd	nd
C20:3n6	nd	nd
C22:1n9	3.51 ± 0.30	nd
C23:0	1.40 ± 0.10	nd
C24:1	1.90 ± 0.10	nd
SFA	29.00 ± 2.00 ^b^	46.56 ± 0.18 ^a^
MUFA	35.01 ± 1.00 ^a^	4.50 ± 0.3 ^b^
PUFA	35.70 ± 0.10 ^b^	49.00 ± 0.5 ^a^
α-Tocopherol	1.29 ± 0.05 ^b^	2.80 ± 0.10 ^a^
β-Tocopherol	nd	nd
γ-Tocopherol	nd	nd
Total tocopherols	1.29 ± 0.05 ^b^	2.80 ± 0.10 ^a^

nd—Not detected. C8:0—caprylic acid; C12:0—lauric acid; C14:0—myristic acid; C15:0—pentadecanoic acid; C16:0—palmitic acid; C16:1—palmitoleic acid; C17:0—heptadecanoic acid; C17:1—heptadecenoic acid; C18:0—stearic acid; C18:1n9t and C18:1n9c—oleic acid (*trans* and *cis*); C18:2n6c—linoleic acid; C18:3n3—α-linolenic acid; C20:0—arachidic acid; C20:1—eicosenoic acid; C20:3n6—dihomo-γ-linolenic acid; C22:1n9—erucic acid; C23:0—tricosylic acid; C24:1—nervonic acid; SFA—saturated fatty acids; MUFA—monounsaturated fatty acids; PUFA—polyunsaturated fatty acids. The results in the same row followed by the same letters do not significantly differ by Tukey’s HSD test (*p* < 0.05).

**Table 3:** Phenolic composition of *A. cicer* and *A. glycyphyllos* flowers (mean ± SD, n = 3).

	*A. cicer* Flowers	*A. glycyphyllos* Flowers
GA (µg/g dw)	965.39 ± 7.02 ^b^	815.25 ± 6.96 ^a^
PTA (µg/g dw)	89.27 ± 5.45 ^b^	330.16 ± 2.40 ^a^
FA (mg/g dw)	1.29 ± 0.01 ^b^	0.55 ± 0.00 ^a^
RUT (mg/g dw)	3.31 ± 0.14 ^b^	6.27 ± 1.13 ^a^
RA (mg/g dw)	1.15 ± 0.85 ^b^	2.37 ± 0.43 ^a^
CNA (mg/g dw)	4.68 ± 0.06 ^b^	0.59 ± 0.00 ^a^
Q (mg/g dw)	0.64 ± 0.02 ^b^	2.78 ± 0.07 ^a^
GENIS (µg/g dw)	42.90 ± 1.94 ^b^	21.33 ± 0.06 ^a^
FORM (µg/g dw)	nd	nd
BIOCH (µg/g dw)	172.45 ± 5.90 ^b^	85.36 ± 0.31 ^a^
TPC (mg GAE/g dw)	5.70 ± 1.72 ^b^	4.91 ± 1.22 ^a^
TFC (mg QE/g dw)	0.45 ± 0.10 ^b^	1.97 ± 0.39 ^a^
TPAC (µg CAE/g dw)	1.22 ± 0.45 ^a^	1.17 ± 0.39 ^a^
TAN (mg CAT/g dw)	0.60 ± 0.10 ^a^	0.50 ± 0.15 ^a^

nd—not detected; GA—gallic acid; PTA—protocatechuic acid; FA—ferulic acid; RUT—rutin; RA—rosmarinic acid; CNA—cinnamic acid; Q—quercetin; GENIS—genistein; FORM—formononetin; BIOCH—biochanin. Means in the same row followed by different letters are significantly different according to Tukey’s honestly significant difference (HSD) test at *p* < 0.05.

**Table 4:** Mineral composition of the flowers of *A. cicer* and *A. glycyphyllos* (mean ± SD, n = 3).

	*A. cicer* Flowers	*A. glycyphyllos* Flowers
K (mg/g dw)	18.45 ± 1.67 ^a^	19.68 ± 1.02 ^a^
Na (mg/g dw)	2.28 ± 0.65 ^a^	1.87 ± 0.77 ^a^
Ca (mg/g dw)	7.02 ± 0.99 ^a^	6.58 ± 0.74 ^a^
Mg (mg/g dw)	2.86 ± 0.86 ^a^	3.25 ± 0.34 ^a^
Zn (µg/g dw)	2.08 ± 0.45 ^a^	2.55 ± 0.25 ^a^
Fe (µg/g dw)	5.16 ± 0.89 ^a^	5.99 ± 0.65 ^a^
Cd (µg/g dw)	nd	nd
Pb (µg/g dw)	nd	nd

nd—Not detected. Means in the same row followed by different letters are significantly different according to Tukey’s honestly significant difference (HSD) test at *p* < 0.05.

**Table 5:** Antioxidant and antibacterial activity of the flowers of *A. cicer* and *A. glycyphyllos* (mean ± SD, n = 3).

	*A. cicer* Flowers	*A. glycyphyllos* Flowers
DPPH (mg TE/g dw)	2.35 ± 0.04 ^a^	2.31 ± 0.06 ^a^
ABTS (mg TE/g dw)	1.82 ± 0.01 ^b^	1.94 ± 0.03 ^a^
FRAP (mg Fe^2+^/g dw)	9.21 ± 1.78 ^a^	7.90 ± 0.95 ^b^
CUPRAC (mg AA/g dw)	73.68 ± 3.81 ^a^	69.79 ± 4.25 ^a^
*S. aureus* ATCC6538	14	12
*E. coli* ATCC8739	0	0
*Ampicilin*	32	25

Means in the same row followed by different letters are significantly different according to Tukey’s honestly significant difference (HSD) test at *p* < 0.05.

## Data Availability

The original contributions presented in this study are included in the article. Further inquiries can be directed to the corresponding authors.

## Supplementary Materials

The following supporting information can be downloaded at https://www.mdpi.com/article/10.3390/plants15192922/s1, Table S1: Validation parameters of the calibration curves for analytes quantified in this study (n = 3).

## Abbreviations

The following abbreviations are used in this manuscript:

ABTS	2,2′-Azino-bis(3-ethylbenzothiazoline-6-sulfonic acid
CUPRAC	Cupric-reducing antioxidant capacity
DPPH	2,2-Diphenyl-1-picrylhydrazyl
FRAP	Ferric-reducing antioxidant power
LOD	Limits of detection
LOQ	Limits of quantification
HPLC	High performance liquid chromatograph
GA	Gallic acid
PTA	Protocatechuic acid
FA	Ferulic acid
RA	Rosmarinic acid
CNA	Cinnamic acid
RUT	Rutin
Q	Quercetin
GENIS	Genistein
FORM	Formononetin
BIOCH	Biochanin
SFA	Saturated fatty acids
MUFA	Monounsaturated fatty acids
PUFA	Polyunsaturated fatty acids
TPC	Total phenolic content
TFC	Total flavonoid content
TPAC	Total phenolic acid content
TAN	Tannin content
HAT	Proceeding via hydrogen atom transfer
SET	Single electron transfer
HSD	Tukey’s honestly significant difference test
