# Supplementary material for: Astragalus cicer L. and Astragalus glycyphyllos L. Flowers as Potential Functional Foods: Nutritional Composition, Chemical Characterization, and Biological Activity
Source: Plants (Basel). 2026 Sep 24;15(19):2922. doi: 10.3390/plants15192922 (PMC13667206; doi:10.3390/plants15192922)
Supplement: Supplementary file 1 [file plants-15-02922-s001.zip › plants-4526678-supplementary.pdf]

Table S1. Validation parameters of the calibration curves for analytes quantified in this study (n=3).

Analytes	Regression equation [a]	Linearity (µg/mL)	R^2	LODs (µg/mL)	LOQs (µg/mL)	Recovery (%)
**GA**	y = 12453x - 38499	55.3-263.0	0.987	2.7	9.4	98.76
**PTA**	y = 53345x - 24884	52.5-261.3	0.965	3.2	13.7	95.89
**FA**	y = 38447x + 88492	55.8-283.2	0.976	2.6	9.2	94.34
**CNA**	y = 39942x + 74638	46.3-248.8	0.993	2.8	12.3	96..21
**RA**	y = 50223x + 64997	50.1-258.8	0.995	3.0	11.8	96.35
**RUT**	y = 27994x - 49343	56.9-262.6	0.989	2.2	7.4	101.45
**Q**	y = 19097x - 29664	56.4-279.3	0.984	2.5	9.7	93.63
**GEMIS**	y = 20686x + 47677	48.5-284.7	0.997	2.3	8.2	92.92
**FORM**	y = 44721x + 68366	50.1-288.4	0.998	3.7	14.8	95.22
**BIOCH**	y = 39234x - 71167	54.3-288.2	0.996	2.9	10.0	105.32

[a] y is the peak area; x refers to the concentration of compounds (µg/mL)
